# Prevalence and associated factors of non-variceal upper gastrointestinal bleeding among cirrhotic patients with upper gastrointestinal bleeding on follow-up at a Tertiary Hospital in Ethiopia

**DOI:** 10.1371/journal.pone.0343977

**Published:** 2026-03-06

**Authors:** Mahlet Mitiku Desalegn, Henok Fisseha, Makida Girma Altaye, Kaleb Assefa Berhane, Wasihun Zerfu Zewde

**Affiliations:** 1 Department of Internal Medicine, St. Paul’s Hospital Millennium Medical College, Addis Ababa, Ethiopia; 2 Department of Internal Medicine, Adera Medical Center, Addis Ababa, Ethiopia; Dalin Tzu Chi Hospital, Buddhist Tzu Chi Medical Foundation, TAIWAN

## Abstract

**Background:**

Liver cirrhosis is a major cause of morbidity and mortality worldwide. Non-variceal upper gastrointestinal bleeding (NVUIGB) accounts for 24–42% of bleeding episodes in cirrhotic patients and carries a mortality rate of 15–30%. Understanding its prevalence and associated factors is critical for prevention and improved outcomes. This study assessed the prevalence and predictors of NVUGIB among cirrhotic patients with upper gastrointestinal bleeding at a tertiary hospital in Addis Ababa, Ethiopia.

**Methods:**

A hospital based cross-sectional study was conducted from December 2020 to December 2023. A total of 234 patients were included in the study. The collected data were analyzed using Statistical Package for Social Sciences (SPSS) version 26.0. Bivariable and multivariable logistic regression was used to assess association between dependent and independent variables. Adjusted odds ratio with a 95% confidence interval was used to estimate the strength of association and level of statistical significance was declared at p value <0.05.

**Results:**

The prevalence of non-variceal upper gastrointestinal bleeding was 31.6% (95% CI: 26.0–37.8). with a mean (±SD) age of 39 ± 14 years. Independent predictors of non-variceal upper gastrointestinal bleeding included longer duration of cirrhosis (AOR = 1.01, 95% CI: 1.001–1.019, p = 0.03) and Human Immunodeficiency Virus (HIV) (AOR = 51.72, 95% CI: 5.65–471.8, p < 0.001). Lower odds of non-variceal upper gastrointestinal bleeding were observed in older patients (AOR = 0.96, 95% CI: 0.93–0.99, p = 0.007), those with hepatitis C virus (AOR = 0.12, 95% CI: 0.05–0.66, p = 0.009) or schistosomiasis (AOR = 0.03, 95% CI: 0.006–0.19, p < 0.001) as the cause of cirrhosis, prior beta-blocker use (AOR = 0.32, 95% CI: 0.14–0.70, p = 0.005), and higher international normalized ratio (AOR = 0.57, 95% CI: 0.36–0.89, p = 0.014). Non-variceal upper gastrointestinal bleeding patients also had lower systolic blood pressure at presentation (AOR = 0.97, 95% CI: 0.95–0.99, p = 0.010) but required fewer blood product transfusions (AOR = 0.25, 95% CI: 0.08–0.71, p = 0.009).

**Conclusion:**

Non-variceal upper gastrointestinal bleeding accounted for nearly one-third (31.6%) of bleeding cases among cirrhosis patients with upper gastrointestinal bleeding. It was significantly associated with longer duration of cirrhosis, younger age, and the presence of comorbidities such as HIV. Targeted screening and preventive measures for high-risk patients may reduce the burden of NVUGIB.

## Background

Liver cirrhosis is a significant cause of morbidity and mortality, being the 14th most common cause of death worldwide [1]. It is characterized by fibrosis and nodule formation of the liver secondary to chronic injury, leading to alteration of the normal lobular organization of the liver. It has different etiologies including viral hepatitis (hepatitis B, hepatitis C), alcoholic liver disease, Non-alcoholic steatohepatitis, autoimmune, toxic, metabolic, genetic and other causes [1,2].

Gastrointestinal (GI) bleeding is one complication among patients with cirrhosis, which is a significant cause of morbidity and mortality. Upper gastrointestinal bleeding (UGIB) refers to a source of bleeding proximal to the ligament of Treitz [3]. It might present as hematemesis or hematochezia with unstable hemodynamic parameters, or as chronic blood loss with melena. GI bleeding accounts for up to 15–30% of overall mortality in patients with cirrhosis [4].

In patients with Cirrhosis, GI bleeding can be classified as variceal or non-variceal bleeding based on the source of the blood loss. Non-variceal upper gastrointestinal bleeding (NVUGIB) is bleeding that develops in the esophagus, stomach or proximal duodenum, and it accounts for about 24–42% of bleeding episodes in cirrhotic patients, and majority of this is due to gastroduodenal ulcers. Other causes include esophagitis, portal hypertensive gastropathy (PHG), gastric vascular ectasia, Mallory-Weiss tear, gastro-duodenal erosions, Dieulafoy’s lesions and others [5,6]. In most studies, Child-Turcotte-Pugh (CTP) class A and CTP class B cirrhosis were shown to have increased incidence of NVUGIB [6,7], whereas in some studies advanced liver disease with CTP class C was found to be associated with increased risk of bleeding, especially with ulcers [8].

In patients with NVUGIB the common risk factors identified are, consumption of Non-steroidal anti-inflammatory drugs (NSAIDS), hypoalbuminemia, presence of significant ascites, advanced liver disease and increased International normalized ratio (INR), and alcohol consumption [9–11].

Endoscopy remains the cornerstone for diagnosing and managing UGIB. Treatment may include endoscopic interventions, proton pump inhibitors (PPIs), vasoactive agents, and supportive measures [12–14].

In Ethiopia, Liver cirrhosis is the seventh leading cause of death, accounting for about 24 deaths per 100,000 populations, with Hepatitis B virus (HBV), alcohol, and hepatitis C virus (HCV) being the three most common etiologies accounting for a pooled estimate of 40.0%, 17.0% and 15.0%, respectively [15]. While studies have explored overall UGIB patterns in Ethiopia, there is a paucity of data on NVUGIB specifically among cirrhotic patients. This study aimed to assess the prevalence of NVUGIB and associated factors among cirrhosis patients with UGIB on follow-up at a tertiary care hospital in Addis Ababa, Ethiopia.

## Materials and methods

### Study design, setting, and participants

A cross-sectional study was conducted among adult cirrhotic patients (aged ≥18 years) presenting with UGIB between December 2020 to December 2023 at the gastroenterology and hepatology unit of St. Paul’s Hospital Millennium Medical College (SPHMMC), a major governmental tertiary care hospital in, Addis Ababa, Ethiopia. The unit provides specialized care for GI and liver diseases. Patients were excluded if they had incomplete clinical or imaging data confirming cirrhosis, recurrent bleeding episodes after the initial presentation, or had not undergone endoscopy.

The required sample size was calculated using the single population proportion formula, assuming a prevalence of 50% to obtain the maximum sample size, as no prior study had estimated the prevalence of non-variceal bleeding among cirrhosis patients with UGIB, 95% confidence level, and 5% margin of error, yielding 384. Given that the total number of cirrhosis patients with UGIB in the hospital registry was 592 (N < 10,000), the finite population correction formula yielded 232.

### Data collection methods and procedures

Data were collected between 01/07/2024, and 01/08/2024 using a pretested checklist designed to capture socio-demographic characteristics, baseline clinical and laboratory parameters, abdominal ultrasound and endoscopic findings, as well as treatment-related variables. Confidentiality of individual patient information was maintained by using code numbers instead of other identifiers, and the information obtained from the chart was used solely for research purposes

Three data collectors, comprising general practitioners and resident physician from SPHMMC, received training on the checklist components, while one individual was trained in data entry procedures. Prior to data entry, patient records were reviewed for completeness, and the first author conducted thorough checks to ensure data consistency and accuracy. The classification of endoscopic findings and determination of bleeding causes were performed according to standard diagnostic criteria to minimize subjectivity among endoscopists.

### Data processing and analysis

Collected data were manually verified for completeness before being entered into Statistical Package for the Social Sciences (SPSS) version 26.0 for analysis. Categorical variables were summarized as frequencies and percentages and compared using the chi-square test. Continuous variables were expressed as means with standard deviations or medians with interquartile ranges, as appropriate and analyzed using binary logistic regression. Missing data for most covariates included in the multivariable logistic regression model were <5% and were handled using simple imputation. Given the low proportion of missingness, sensitivity analyses were not performed for these variables. Total bilirubin levels had a higher proportion of missing data (7%); therefore, a sensitivity analysis was conducted. The analysis suggested that missingness was at random, and the results of the multivariable model remained materially unchanged.

Bivariate logistic regression analysis was performed to identify candidate variables with p-values ≤ 0.20, which were subsequently included in multivariate logistic regression models to determine factors independently associated with the prevalence of NVUGIB among cirrhosis patients. Adjusted odds ratios (AORs) with 95% confidence intervals (CIs) were calculated to estimate the strength of associations, and statistical significance was set at p < 0.05.

### Ethical considerations

Ethical approval for this study was obtained from the Institutional Review Board of SPHMMC (Approval Number: PM23/948). As this was a retrospective chart review, informed consent was waived by the ethics committee. Patient identifiers were anonymized to ensure confidentiality, and all data were analyzed anonymously and used solely for research purposes. The study was conducted in accordance with the ethical standards of the Declaration of Helsinki and the Belmont Report.

### Operational definition

Liver cirrhosis: was diagnosed based on the presence of two or more of the following:

- Clinical signs of cirrhosis (jaundice, ascites, caput medusa, clubbing, palmar erythema, spider naevi, gynecomastia, female pubic hair pattern, encephalopathy, splenomegaly, or asterixis)
- Impaired liver function test consistent with cirrhosis (INR ≥ 1.5 and serum albumin ≤ 3.4 gm/dl)
- Imaging diagnosis of cirrhosis (surface nodularity, coarse and heterogeneous echotexture/ attenuation, segmental atrophy or hypertrophy, ascites, splenomegaly, gall bladder wall thickening, or portal vein diameter >13 mm on abdominal ultrasound and/or CT scan)
- Transient elastography (FibroScan^®^) > 12.5 Kilopaskal (KPa)
- AST (aspartate aminotransferase) to Platelet Ratio Index (APRI) score of ≥ 1.5 or Fibrosis-4 (FIB-4) score ≥ 3.25 [16].

## Results

A total of 646 patients with cirrhosis attended the gastroenterology and hepatology clinic at SPHMMC during the study period. Among these, 255 patients (39.4%) presented with UGIB. Of the UGIB cases, 21 patients were excluded due to incomplete documentation, resulting in a final sample of 234 cirrhotic patients with UGIB.

### Sociodemographic results

The mean age of the 234 patients was 40 ± 14 years (range: 18–87 years), with males comprising 158 patients (67.5%). The mean age was 39 ± 14 years among patients with NVUGIB and 41 ± 14 years among those with variceal UGIB. Males accounted for 70.3% of NVUGIB and 66.3% of variceal UGIB cases.

### Clinical characteristics of cirrhotic patients with UGIB

Hepatitis B virus was the most common etiology of cirrhosis identified in patients NVUGIB (48.6%), followed by HCV (16.2%), and alcohol-associated liver disease (13.5%), wherein patients with variceal UGIB, the most common causes were HBV (27.5%), followed by Alcohol-associated cirrhosis (21.3%) and HCV (15.0%). Hepatosplenic schistosomiasis was significantly less frequent in the NVUGIB group (2.7%) compared to the variceal UGIB group (21.3%). Additionally, hepatocellular carcinoma (HCC) was identified in approximately 11.3% of patients with variceal UGIB and 13.5% of those with NVUGIB.

In assessing the severity of cirrhosis, CTP Class A was relatively uncommon in both groups but slightly more frequent in the NVUGIB group (8.1%) compared to the variceal UGIB group (3.8%). Similarly, CTP Class B was more prevalent among NVUGIB patients (37.8%) than those with variceal UGIB (30.0%). Conversely, CTP Class C cirrhosis was more common in the variceal UGIB group (66.3%) than in the NVUGIB group (54.1%). Despite these differences, CTP Class C was the predominant category in both groups.

Regarding comorbidities, a slightly higher number of patients had Human immunodeficiency virus (HIV) in NVUGIB group (6.8%) compared to the Variceal UGIB group (3.1%), whereas more patients had chronic kidney disease (CKD) in the variceal group (10.0%) compared to patients with NVUGIB (5.4%). Prevalence of hypertension (HTN) was comparable in both groups.

Assessment of prior medication use revealed that NSAID consumption was common and comparable between the two groups, with 55.0% in the variceal UGIB group and 56.8% in the NVUGIB group. Beta-blocker use was notably higher in the variceal UGIB group (47.5%) compared to the NVUGIB group (24.3%). Proton pump inhibitor (PPI) use was relatively low in both groups but slightly more frequent in the variceal UGIB group (8.8%) than in the NVUGIB group (5.4%). Aspirin use was identified in approximately 2.7% of patients in the NVUGIB group (Table 1).

**Table 1:** Clinical characteristics of cirrhotic patients with UGIB at SPHMMC, Addis Ababa, Ethiopia, 2020 to 2023 (N = 234).

		Study Participants		Variceal UGIB		NVUGIB		P-Value
Variable	Category	Total (n)	Percent (%)	Count (n)	Percent (%)	Count (n)	Percent (%)	
Cause of Cirrhosis	HBV	80	34.2	44	27.5%	36	48.6%	.057
	HCV	36	15.4	24	15.0%	12	16.2%	.013
	Alcohol	44	18.8	34	21.3%	10	13.5%	.085
	MASLD	0	0	0	0.0%	0	0.0%	
	Schistosomiasis	36	15.4	34	21.3%	2	2.7%	.024
	Others	38	16.2	24	15.0%	14	18.9%	.997
Severity of Cirrhosis	CTP Class A	12	5.1	6	3.8%	6	8.1%	.143
	CTP Class B	76	32.5	48	30.0%	28	37.8%	.071
	CTP Class C	146	62.4	106	66.3%	40	54.1%	.054
Comorbidities	DM	12	5.1	8	5.0%	4	5.4%	.56
	HTN	16	6.8	11	6.9%	5	6.8%	.607
	HF	2	0.8	2	1.3%	0	0.0%	.467
	CKD	20	8.5	16	10.0%	4	5.4%	.181
	HIV	10	4.3	5	3.1%	5	6.8%	.175
	Others	18	7.6	14	8.8%	4	5.4%	.271
	HCC	28	12	18	11.3%	10	13.5%	.383
Previous GI bleeding	No	182	77.8	122	76.3%	60	81.1%	
	Yes	52	22.2	38	23.8%	14	18.9%	.499
Prior medication use	NSAIDS	130	55.5	88	55.0%	42	56.8%	.457
	Aspirin	2	0.8	0	0.0%	2	2.7%	.094
	Beta-blockers	94	40.1	76	47.5%	18	24.3%	.001
	Diuretics	117	50.0	81	50.6%	36	48.6%	.444
	PPI	18	7.6	14	8.8%	4	5.4%	.271
	Others	40	17.1	24	15.0%	16	21.6%	.409
Duration of cirrhosis	Mean	21		18		26		.08
	SD	37		25		54		
MELD score	Mean	17		17		17		.732
	SD	7		7		7		
Systolic blood pressure	Mean	114		116		111		.110
	SD	19		19		18		
Diastolic blood pressure	Mean	71		71		70		.751
	SD	13		13		14		

UGIB: Upper Gastrointestinal Bleeding, NVUGIB: Non-Variceal Upper Gastrointestinal Bleeding, HBV: Hepatitis B virus, HCV: Hepatitis C virus, MASLD: Metabolic dysfunction-associated steatotic liver disease, CTP: Child Turcotte Pugh, DM: Diabetes Mellitus, HTN: Hypertension, HF: Heart Failure, CKD: Chronic Kidney Disease, HIV: Human Immunodeficiency Virus, HCC: Hepatocellular Carcinoma, NSAIDs: Non-steroidal Anti-inflammatory drugs, PPI: Proton pump inhibitors, SD: Standard deviation, MELD: Model for End-stage Liver Disease, SPHMMC: St. Paul’s Hospital Millennium Medical College.

Approximately one-fourth (23.8%) of patients with variceal UGIB had a prior history of GI bleeding, compared to 18.9% in the NVUGIB group.

The duration of cirrhosis showed wide variability in both groups, with a median duration of 26 months (IQR: 22) in the NVUGIB group and 18 months (IQR: 12) in the variceal UGIB group. The MELD scores were similar between the two groups. Median systolic blood pressure at presentation was also comparable, recorded as 112 mmHg (SD 18) in the NVUGIB group and 112 mmHg (SD 19) in the variceal UGIB group (Table 1).

**Table 2:** Laboratory parameters of cirrhotic patients with UGIB at SPHMMC, Addis Ababa, Ethiopia, 2020 to 2023 (N = 234).

Variable	Study Participants		Variceal UGIB		NVUGIB			
	Mean	SD	Mean	SD	Mean	SD	P-value	Missing
WBC (/mm^3^)	6185	4274	6028	4455	6525	3861	.409	<.5%
Post bleeding Hb	11.2	3.1	10.9	3.4	11.9	2.2	.022	<.5%
Platelets (/mm^3^)	129376.1	98082.6	124362.5	98310.0	140216.2	97367.9	.251	<.5%
AST Value	109.05	173.55	105.79	189.98	116.11	132.06	.673	<.5%
ALT Value	63.5	136.7	67.3	160.6	55.2	58.0	.541	<.5%
ALP Value	181.6	153.9	171.1	142.5	204.3	175.1	.602	<.5%
TB Value	2.691	3.434	2.698	3.535	2.676	3.226	.849	7%
Albumin Value	2.54	.76	2.57	.76	2.49	.76	.48	1%
INR	2.01	.99	2.08	1.00	1.84	.93	.070	<.5%
Creatinine	1.01	.62	1.00	.62	1.04	.63	.680	<.5%
Serum Sodium	135.3	6.8	135.7	6.6	134.5	7.1	.20	1.1%

UGIB: Upper Gastrointestinal Bleeding, NVUGIB: Non-Variceal Upper Gastrointestinal Bleeding, WBC: White Blood cell, AST: Aspartate Aminotransferase, ALT: Alanine Aminotransferase, ALP: Alkaline Phosphatase, Hb: Hemoglobin TB: Total bilirubin, INR: International Normalized Ratio, SPHMMC: St. Paul’s Hospital Millennium Medical College.

### Laboratory parameters

The mean white blood cell (WBC) count was 6,525 cells/mm³ (SD 3,864) in patients with NVUGIB and 6,028 cells/mm³ (SD 4,455) in the variceal UGIB group. Post-bleeding hemoglobin (Hb) levels averaged 11.9 g/dL (SD 2.2) in the NVUGIB group and 10.9 g/dL (SD 3.1) in the variceal group. The mean INR was 1.84 (SD 0.93) among NVUGIB patients, compared to 2.08 (SD 1.0) in those with variceal bleeding. Serum creatinine levels were similar between groups, with a mean of 1.04 mg/dL (SD 0.63) in NVUGIB patients and comparable values in the variceal group (Table 2).

### Endoscopic findings and causes of UGIB in cirrhotic patients

**Fig 1:**
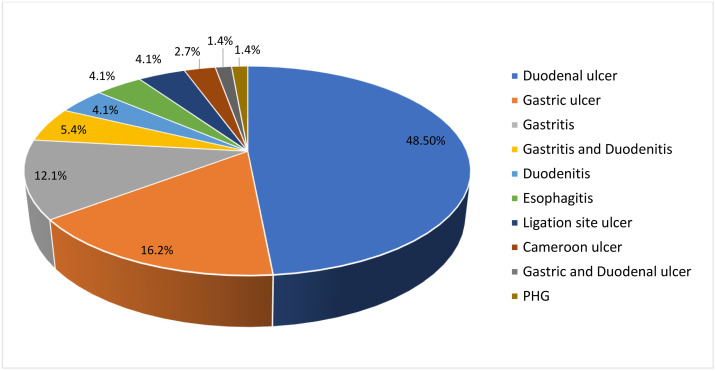
Causes of non-variceal bleeding among cirrhotic patients with upper gastrointestinal bleeding at SPHMMC, Addis Ababa, Ethiopia (2020–2023, N = 74).

Among the cirrhotic patients presenting with UGIB, the prevalence of NVUGIB was 31.6% (95% CI 26% − 37.8%), and the majority of the remaining patients had variceal bleeding (67.1%), and 1.3% of the patients had normal Endoscopic findings. The most common etiology identified was duodenal ulcer, accounting for 48.5% of all NVUGIB cases, followed by gastric ulcer (16.2%), gastritis (12.1%), and both gastritis and duodenitis (5.4%). Less frequent causes included duodenitis (4.1%), esophagitis (4.1%), ligation site ulcer (4.1%), Cameroon ulcer (2.7%), gastric and duodenal ulcer (1.4%), and PHG (1.4%), collectively accounting for the remaining 17.2% of the cases (Fig 1).

### Interventions and outcomes

Approximately one in four patients (26.5%) presenting with UGIB required blood product transfusions. Endoscopic interventions were performed in over half of the patients (57.7%, n = 135). Overall mortality was 17.5%, with 20.3% in the NVUGIB group and 17.5% in the variceal UGIB group. The highest mortality was observed in patients with duodenal ulcers (29.3%), followed by gastritis (9.7%) (Table 3).

**Table 3:** Interventions and outcomes of cirrhotic patients with UGIB at SPHMMC, Addis Ababa, Ethiopia, 2020 to 2023 (N = 234).

		Study Participants		Variceal UGIB		NVUGIB		
Variable	Category	Total (n)	Percent (%)	Count (n)	Percent (%)	Count (n)	Percent (%)	P value
Intervention	Endoscopic	150	57.7	106	66.3%	44	59.5%	.001
	Blood transfusion	62	26.5	50	31.3%	12	16.2%	.015
Treatment outcome	Alive	193	82.48%	134	83.8%	59	79.7%	.452
	Dead	41	17.5%	26	16.3%	15	20.3%	

UGIB: Upper Gastrointestinal Bleeding, NVUGIB: Non-Variceal Upper Gastrointestinal Bleeding, SPHMMC: St. Paul’s Hospital Millennium Medical College.

### Factors associated with NVUGIB

Binary logistic regression analysis identified several factors independently associated with NVUGIB among cirrhosis patients. A longer duration of cirrhosis (p = 0.03, AOR = 1.01, 95% CI [1.001–1.019]) and the presence of HIV as a comorbid condition (p < 0.001, AOR = 51.72, 95% CI [5.65–471.8]) were strong predictors of NVUGIB. In contrast, older age (p = 0.007, AOR = 0.96, 95% CI [0.93–0.99]), hepatitis C virus infection (p = 0.009, AOR = 0.12, 95% CI [0.05–0.66]), and schistosomiasis (p < 0.001, AOR = 0.03, 95% CI [0.01–0.19]) as underlying causes of cirrhosis were associated with a lower likelihood of NVUGIB compared to variceal bleeding. Prior beta-blocker use (p = 0.005, AOR = 0.32, 95% CI [0.14–0.70]) and higher INR values (p = 0.014, AOR = 0.57, 95% CI [0.36–0.89]) were also linked to a reduced risk of NVUGIB. Patients with NVUGIB presented with lower systolic blood pressure (p = 0.010, AOR = 0.97, 95% CI [0.95–0.99]) but required fewer blood product transfusions (p = 0.009, AOR = 0.25, 95% CI [0.08–0.71]) compared to those with variceal bleeding. CTP class showed no significant association with NVUGIB (Table 4).

**Table 4:** Multivariable binary logistic regression analysis of cirrhotic patients with UGIB at SPHMMC, Addis Ababa, Ethiopia, 2020 to 2023 (N = 234).

				95% C.I.		
Categories	Variables	OR	AOR	Lower	Upper	P value
Demographics	Age	.98	.963	.937	.990	**.007**
Cause of Cirrhosis	HBV	1.00	1.00			
	HCV	.61	.188	.053	.661	**.009**
	ALD	.35	.379	.140	1.024	.056
	Schistosomiasis	.07	.034	.006	.193	**.001**
	Others	.71	.507	.187	1.379	.184
Severity of cirrhosis	CTP Class A	1.00	1.00			.166
	CTP Class B	.58	.261	.049	1.389	.115
	CTP Class C	.38	.191	.034	1.064	.059
Clinical parameters	Systolic Blood Pressure	.98	.973	.953	.993	**.010**
	Duration of Cirrhosis	1.01	1.010	1.001	1.019	**.037**
	Beta-blockers	2.81	.316	.142	.706	**.005**
	Aspirin			.000		.999
	Blood Transfusion	2.35	.251	.088	.713	**.009**
Laboratory parameters	Post-bleeding Hemoglobin	1.12	1.120	.980	1.279	.096
	INR	.76	.570	.363	.893	**.014**
	Serum Sodium	0.97	.959	.906	1.015	.149
Comorbidities	CKD	1.94	.547	.129	2.311	.411
	HIV	99	51.720	5.669	471.826	**.001**

HBV: Hepatitis B Virus, HCV: Hepatitis C virus, CTP: Child Turcotte Pugh, INR: International Normalized Ratio, CKD: Chronic Kidney Disease, HIV: Human Immunodeficiency Virus, SPHMMC: St. Paul’s Hospital Millennium Medical College, UGIB: Upper Gastrointestinal Bleeding.

## Discussion

In this study, NVUGIB occurred in 31.6% of cirrhotic patients with UGIB, slightly higher than reported in India (24.5%) [6] but lower than in Pakistan (56.93%) [17] and Thailand (51%) [18]. The lower prevalence compared to the latter studies may relate to delayed endoscopy in our cohort, potentially allowing ulcer healing, as well as our older patient population, given the observed negative association between NVUGIB and age. Differences in inclusion criteria, such as the exclusion of recent variceal interventions in the Pakistani study, may also explain the variation.

Peptic ulcer disease (gastric and/or duodenal ulcers) was found to be the most common etiology of NVUGIB in our findings, which was present in around two-third of our patients (66.1%). Although at a relatively higher prevalence in our study, the finding of peptic ulcer causing the majority of NVUGIB in cirrhosis patients is similar with most studies such as the Czech study of 108 patients [19] and the Indian study of 550 patients which reported incidence of 40.5% of gastroduodenal ulcers [6]. This relatively higher number in our study might in part be associated with the high prevalence of Helicobacter pylori (H. Pylori) in our country, which is reported in 52.2% in a meta-analysis of 18,890 patients [20]. Less common etiologies such as PHG is found in only 1.4% of our study, which may show PHG is an uncommon cause of acute UGIB among cirrhotic patients, as supported by other studies which reported an incidence between 2 and 12%, albeit it plays a major role in the pathophysiology of ulcer formation and poor gastric mucosal healing [7,21].

Longer duration of cirrhosis was shown to have a strong association with NVUGIB in our study with mean duration found to be 24±.5 years. Longer duration of cirrhosis is correlated with increased severity of cirrhosis, and with the development and progression of portal hypertension and PHG [22]. Considering the pathophysiology of NVUGIB including peptic ulcer, mechanisms suggested in liver cirrhosis include portal hypertension-induced impairment of the gastric mucosal defenses, reduced prostaglandins, decreased gastric acid secretion, elevated serum gastrin concentration, impaired mucus secretion, a reduction in potential difference of the gastric mucosa [23,24].

Certain comorbidities such as HIV are also identified as strong predictors of NVUGIB in our findings. This might be a result of multiple factors, such as the presence of opportunistic infections such as Herpes simplex virus (HSV), cytomegalovirus (CMV), typical or atypical mycobacterium infections that are ulcerogenic or causing esophagitis, as stated in literatures [25].

Lower INR values were seen with NVUGIB in our study, than the higher values seen with the variceal group. This finding is different from the study done in china that compared variceal UGIB with peptic ulcer bleeding, which found higher INR, bilirubin, and creatinine values in non-variceal groups [13]. This may be in part that higher INR values might implicate a worse decompensation of liver function, which is one factor for the development of varices. It may also be due to other etiologies in NVUGIB that were not addressed in their study. In addition, although there was a slightly increased number of patients with HCC in the NVUGIB, it was not statistically significant, unlike the study in china.

Schistosomiasis was a less common cause of liver disease in this study. The main pathophysiologic mechanism of developing UGIB in schistosomiasis is the causation of pre-sinusoidal portal hypertension, which in turn will result in increased splanchnic vasodilation and formation of varices [26]. Therefore, variceal bleeding is the more common presentation of UGIB, as shown in our study with the significant negative association with NVUGIB.

The incidence of NVUGIB in cirrhotic patients had significant negative association with the use of beta-blockers in our study, compared to variceal groups. This may be due to that patients are started on beta-blockers for primary or secondary prophylaxis for variceal bleeding from prior screening with presence of varices or prior episodes of bleeding, hence, this increased incidence of varices may predispose them to variceal UGIB than non-variceal [13]. Although NSAID use is generally contraindicated in patients with decompensated cirrhosis (CTP class B and C) due to the risk of renal impairment, GI bleeding, and worsening ascites, we observed a relatively high prevalence of NSAID use in our cohort. This may be attributed to factors such as the over-the-counter availability of NSAIDs without prescription, limited access to safer alternative analgesics, and inadequate patient awareness regarding the associated risks.

In addition, patients with NVUGIB were less likely to require blood product transfusions, similar to the study done in china [13]. Mortality was 17.5% in our patients with UGIB, and 20.3% in those with NVUGIB, which we did not find to differ significantly among our two groups of patients. Relatively similar mortality rates were recorded in other studies, of mortality 23.5% − 25% [3,27].

To the best of the authors’ knowledge, this is the first study of its kind in the country, providing valuable baseline data for future research and comparative analyses. However, his study has several limitations. Delayed access to endoscopy during acute bleeding episodes may have led to misclassification or underestimation of NVUGIB, as some lesions may have healed by the time of evaluation. Although the small number of HIV-positive patients (n = 10) in our study resulted in wide confidence intervals and reduced statistical precision, sensitivity analyses showed that inclusion or exclusion of the HIV variable did not materially alter the study findings. The retrospective design and reliance on medical records may have introduced information bias, including incomplete medication histories, laboratory data, and inconsistent *Helicobacter pylori* testing, which may partly explain the high prevalence of peptic ulcer disease. Residual confounding cannot be excluded, and causal inferences cannot be made. Future prospective registry based studies with timely endoscopy are recommended to minimize this limitations and more accurately define the true burden and determinants of NVUGIB among cirrhotic patients presenting with UGIB.

## Conclusion

The prevalence of NVUGIB among patients with cirrhosis in this study was 31.6%. A longer duration of cirrhosis, younger age, and HIV co-infection were strongly associated with an increased likelihood of NVUGIB, whereas prior use of beta-blockers and higher INR, hepatitis C virus or schistosomiasis as a cause of cirrhosis were associated with a reduced risk. Early identification of high-risk patients and preventive strategies are essential to reduce NVUGIB burden and improve outcomes. Future research should explore predictive models and larger sample sizes to strengthen associations.

## Supporting information

S1 File STROBE-checklist-v4-combined-PlosMedicine [1]. (DOCX)
